# Regulation of Immune Cell Function by PPARs and the Connection with Metabolic and Neurodegenerative Diseases

**DOI:** 10.3390/ijms19061575

**Published:** 2018-05-25

**Authors:** Gwenaëlle Le Menn, Jaap G. Neels

**Affiliations:** Université Côte d’Azur, Inserm, C3M Nice, France; gwenaelle.le-menn@unice.fr

**Keywords:** obesity, type 2 diabetes, atherosclerosis, neurodegenerative disease, inflammation, macrophages, T cells, PPARs, metabolism, gender

## Abstract

Increasing evidence points towards the existence of a bidirectional interconnection between metabolic disease and neurodegenerative disorders, in which inflammation is linking both together. Activation of members of the peroxisome proliferator-activated receptor (PPAR) family has been shown to have beneficial effects in these interlinked pathologies, and these improvements are often attributed to anti-inflammatory effects of PPAR activation. In this review, we summarize the role of PPARs in immune cell function, with a focus on macrophages and T cells, and how this was shown to contribute to obesity-associated inflammation and insulin resistance, atherosclerosis, and neurodegenerative disorders. We address gender differences as a potential explanation in observed contradictory results, and we highlight PPAR-induced metabolic changes as a potential mechanism of regulation of immune cell function through these nuclear receptors. Together, immune cell-specific activation of PPARs present a promising therapeutic approach to treat both metabolic and neurodegenerative diseases.

## 1. The Interrelationship between Metabolism, Inflammation, and Neurodegenerative Disease

### 1.1. Inflammation and Metabolic Disease

Although inflammation is a vital response to infection and tissue injury, non-resolved chronic inflammation is associated with many pathological processes. Several of these pathologies, in which inflammation is a common denominator, are grouped under metabolic syndrome, including obesity, type 2 diabetes, cardiovascular disease, and fatty liver disease [1].

Over the past two decades, a clear link has been established between obesity-associated inflammation and the development of insulin resistance, which eventually leads to type 2 diabetes [1]. As a result of insulin resistance, the body needs higher levels of insulin to help glucose enter cells. The β cells in the pancreas try to keep up with this increased demand for insulin by producing more. Over time, however, insulin resistance can lead to type 2 diabetes and prediabetes, because the β cells fail to keep up with the body’s increased need for insulin.

Initially, studies showed that adipose tissue expansion in obesity is accompanied by an increase in cytokine and chemokine expression, such as tumor necrosis factor (TNF)-α, interleukin (IL)-6, monocyte chemoattractant protein (MCP)-1, and interferon (IFN)-γ. Some of these cytokines/chemokines were shown to impair insulin action in normally insulin-sensitive tissues, leading to insulin resistance. Later, it was demonstrated that this obesity-induced adipose tissue inflammation was largely the result of a shift in the balance of anti-inflammatory towards pro-inflammatory immune cells [2]. In lean adipose tissue, regulatory B cells (Bregs), regulatory T cells (Tregs), T helper 2 (Th2) cells, eosinophils, and type 2 innate lymphoid cells (ILC2s) maintain an anti-inflammatory environment through the production of IL-10, IL-4, IL-5, and IL-13. These anti-inflammatory cytokines promote anti-inflammatory M2 polarized macrophages in adipose tissue. By contrast, obesity-associated adipose tissue expansion is accompanied by an increase in elastase-secreting neutrophils, mast cells, and IFNγ-secreting CD8^+^ T cells, Th1 cells, and natural killer (NK) cells. Inflammatory mediators secreted by these cells promote pro-inflammatory M1 macrophage polarization and their release of IL-1β, IL-6, and TNF-α cytokines [2].

Likewise, atherosclerosis is also associated with a chronic and non-resolving immune response. The accumulation of lipoproteins in the arterial wall, characteristic of atherosclerosis, triggers first an innate immune response, dominated by monocyte/macrophages, followed by an adaptive immune response involving primarily Th1, but also Th17 and Th2 cells and B cells, alongside a progressive decrease in Tregs [3]. As in adipose tissue, atherosclerotic plaques can contain both inflammatory and resolving macrophages. The pro-inflammatory macrophages secrete cytokines, proteases, and other factors that can cause plaque morphological changes and progression that can eventually trigger plaque rupture, whereas resolving macrophages carry out functions that can suppress plaque progression and promote plaque regression and/or stabilization [3].

### 1.2. Inflammation as a Link between Metabolic Disease and Neurodegenerative Disorders

Both human studies and animal models concur to suggest an interrelationship between metabolic disease and neurodegenerative disorders (NDDs), such as Alzheimer’s disease, Huntington’s disease, Parkinson’s disease, and multiple sclerosis [4,5,6,7,8,9]. Higher body mass index represents a risk factor for the development of these NDDs [4,5,6,7,8,9]. Inflammation might be linking metabolic disease to NDDs, since a growing body of observational and experimental data shows that inflammatory processes, termed neuroinflammation, contribute to the onset and progression of neuronal degeneration [10]. Furthermore, this link between metabolic disease and neuroinflammation goes both ways, since hypothalamic inflammation has been linked to the development and progression of obesity and its sequelae [11,12]. Hypothalamic inflammation induced by obesogenic diets occurs before significant body weight gain, and precedes inflammation in peripheral tissues. This results in the uncoupling of caloric intake and energy expenditure, not only leading to overeating and weight gain, but also contributes to obesity-associated insulin resistance via altered neurocircuit functions. For example, hypothalamic inflammation modulates insulin secretion by pancreatic β cells, adipose tissue lipolysis, and hepatic glucose production [13,14]. Microglia cells, the brain counterpart of macrophages, play a major role in the neuroinflammation observed in both NDDs and the obesity-associated hypothalamic inflammation [10,11]. The aggregates of amyloid β-peptide (Aβ) and α-synuclein, that respectively characterize Alzheimer’s and Parkinson’s disease, have been shown to induce microglia activation, which augments the level of neuroinflammatory mediators, that in turn worsen these NDDs [10]. Likewise, an obesogenic diet leads to an accumulation of activated microglia within the hypothalamus that produce a variety of proinflammatory cytokines [11]. Furthermore, high fat feeding is associated with the accumulation and activation of astrocytes in the hypothalamus, which also produce a variety of inflammatory factors [11]. In Huntington’s disease, expression of mutant Huntingtin (HTT) protein results in a cell-autonomous pro-inflammatory state of activation of microglia and, to a certain extent, of astrocytes [15]. Multiple sclerosis is characterized by the progressive destruction of axon myelin sheaths by the action of autoreactive immune cells (including T cells and macrophages) [10].

**Figure 1:**
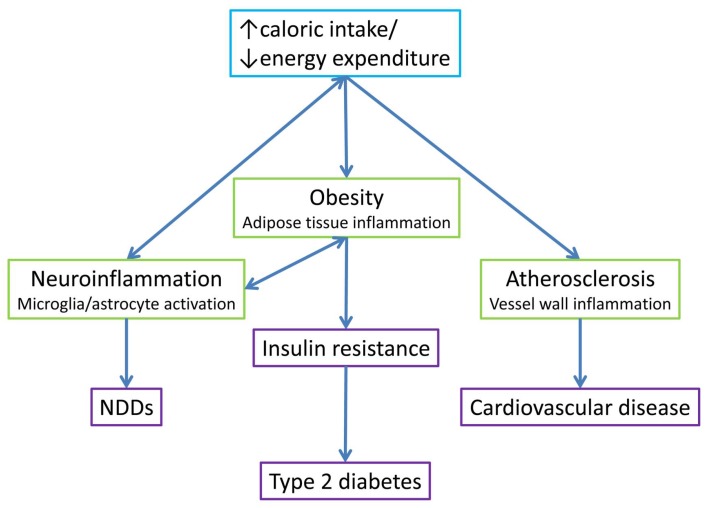
Interconnection between metabolism, inflammation, and neurodegeneration. An imbalance between caloric intake and energy expenditure has been linked to both metabolic disease (obesity and atherosclerosis) and neurodegenerative disorders. These pathologies all have a state of unresolved chronic inflammation in common. The link between neuroinflammation and obesity and associated sequelae is bidirectional, since hypothalamic inflammation leads to uncoupling of caloric intake and energy expenditure, leading to obesity, but also contributes to obesity-induced insulin resistance (and subsequent type 2 diabetes) via altered neurocircuit functions.

Taken together, both animal models and human studies strongly suggest that there is a close interconnection between metabolism, inflammation, and neurodegeneration (see Figure 1). With inflammation as a link between metabolic disease and NDDs, therapies targeting inflammation might both re-establish metabolic homeostasis and have efficacy in counteracting cognitive decline.

## 2. The Role of Metabolism in Immune Cell Function

Glycolysis, oxidative phosphorylation (OXPHOS), glutaminolysis, and/or fatty acid oxidation (FAO) are metabolic pathways that generate energy needed to satisfy basic cellular functions. Regarding immune cells, it was shown over the years that these cells can adapt their metabolism, from one pathway to another, to support the bioenergetically demanding processes of growth and effector function during an immune response.

### 2.1. Adaptive Immune Cells

The first metabolic change encountered by lymphocytes appears upon activation when shifting from quiescent cells with a relatively low metabolism to activated and proliferating cells, that have high metabolic needs. This shift is supported by a switch from an oxidative metabolism towards anaerobic glycolysis (Warburg effect) following antigen recognition by both T and B cells [16,17]. Indeed, lymphocyte activation is accompanied by an elevated glucose uptake through increased translocation of glucose transporter 1 (GLUT1) to the cellular membrane [18,19]. Increase in glutaminolysis in also observed in both cell types as glutamine is an essential substrate for the tricarboxylic acid cycle [20,21]. For B cells, activation is also accompanied by an increased OXPHOS, but data on the metabolic profile of distinct B cell subsets is still lacking [17]. As for T cells, activated CD4^+^ T cells will polarize into different subpopulations with their own inflammatory and metabolic phenotype (Th1, Th2, Th17, and Tregs). Anti-inflammatory Tregs are poorly proliferative, whereas pro-inflammatory T cell subsets can be highly proliferative. In this regard, studies showed that Th1, Th2, and Th17 cells use glycolysis to meet their energy demands, whereas Tregs have high lipid oxidation rates [22,23]. Furthermore, it was demonstrated that by directly manipulating cell metabolism one can regulate CD4^+^ T cell fate; for example, inhibition of glycolysis blocks Th17 development and promotes T cell polarization towards Treg cells [23]. CD8^+^ memory T cells largely depend on FAO for their metabolic needs, and in line with this, carnitine palmitoyltransferase Ia (CPT1a) expression (rate-limiting enzyme of FAO pathway) was found to promote the differentiation into this subpopulation [24].

### 2.2. Innate Immune Cells

Granulocytes, dendritic cells (DC), and M1 type macrophages rely on glucose metabolism upon activation, while M2 macrophages depend on FAO. Unlike lymphocytes, activated myeloid cells tend to be non-proliferative, but still mostly exhibit an increased glycolytic metabolism upon activation, which is essential to acquire their effector function.

Indeed, neutrophil effector functions, such as neutrophil extracellular trap formation, tissue infiltration and phagocytosis, were decreased in the presence of the 2-deoxy-glucose, an inhibitor of glycolysis [25,26]. In a recent study on mast cells, seahorse experiment results showed an increase of glycolysis, as well as OXPHOS, following their activation. The latter was particularly implicated in the degranulation process and cytokine production [27]. As for eosinophil and basophil metabolism, evidence suggests a glycolytic metabolism after their activation, but this needs to be investigated further [28]. DCs shift from naïve DCs, using mainly FAO and OXPHOS metabolism, to glycolysis, upon activation. Increase of glucose metabolism is then mainly implicated in the increase in de novo fatty acid synthesis that seems to correlate with the immunogenic phenotype of DCs [29]. Similar to T cells, macrophage activation can give rise to the polarization into pro-inflammatory M1 or anti-inflammatory M2 macrophages that exhibit metabolic differences. While M1 macrophages preferentially use glycolysis to support the production of inflammatory cytokines, such as IL-1β and TNF-α via the activation of nuclear factor-κB (NF-κB) and activator protein-1 (AP-1) signaling, M2 macrophages use lipid oxidation as energy source [30]. In this case, lipid oxidation is supported by an increase in the expression of fatty acid translocase (FAT)/CD36 and CPT1a, that favors lipid import into cells and mitochondria, respectively [30,31].

It is clear from these findings that metabolism plays an important role in the immune cell fate and inflammatory phenotype. Overall, a distinction can be made between pro-inflammatory cells, that require a rapid burst of energy and macromolecule synthesis via glycolysis to produce cytokines, and quiescent or anti-inflammatory cells, that use mostly oxidation (FAO and OXPHOS) for their survival and longevity. As a consequence, manipulating immune cell metabolism has become an interesting approach to control the immune response.

## 3. Role of PPARs in Immune Cell Function

### 3.1. PPARs and Their Mode of Action

The peroxisome proliferator-activated receptor (PPAR) subfamily of nuclear hormone receptors consist of three different isoforms; PPARα, PPARβ, and PPARγ, that are each expressed in various tissues and cell types, and regulate the transcription of a large variety of genes implicated in metabolism, cell proliferation/differentiation, and inflammation [32]. These different PPAR members have a conserved structure that includes an N-terminal ligand-independent transactivation domain, a DNA binding domain, and a C-terminal ligand-binding domain and ligand-dependent activation domain [33]. This C-terminal region is implicated in receptor heterodimerization with the obligatory transcriptional partner, the retinoid X receptor (RXR). These heterodimers bind to specific DNA sequence elements called peroxisome proliferator response elements (PPREs) in the regulatory region of their target genes. Binding of synthetic or endogenous ligands (fatty acids and their derivatives) induces a conformational switch in the receptors, leading to dissociation of co-repressor proteins and recruitment of co-activator proteins to enhance the transcription of target genes [33]. This direct transcriptional regulation of PPARs through binding to PPREs largely concerns target genes involved in transport, synthesis, storage, mobilization, activation, and oxidation of fatty acids. However, the regulation of immune cell function by PPARs, the topic of this review, is thought to mostly implicate transcription regulation of target genes through indirect mechanisms. The best-known mechanism by which PPARs regulate inflammation is through transrepression [34]. This activity involves indirect association (tethering) of the PPARs with target genes. There are many mechanisms by which PPARs can transrepress inflammatory responses, including competition for a limiting pool of coactivators, direct interaction with the p65 subunit of NF-κB and c-Jun subunit of AP-1, modulation of p38 mitogen-activated protein kinase (MAPK) activity, and partitioning the corepressor B-cell lymphoma 6 (BCL-6) [34].

### 3.2. Role of PPARs in Immune Cells

There is a vast amount of literature (including many excellent reviews) on the anti-inflammatory roles of the different PPARs in a multitude of inflammatory diseases (for selection of reviews, see [32,35,36,37,38,39,40,41,42,43,44,45,46,47,48,49]). Many of these studies were performed in global knockout models and/or PPAR agonists/antagonists were administered systemically. The global/systemic nature of these latter studies often does not allow for the interpretation of the role of PPARs in specific immune cells, since the effects observed could be due to numerous PPAR actions unrelated to their function in immune cells. Furthermore, several studies treated immune cells with endogenous PPAR ligands that are also known to have PPAR-independent effects, so again, this complicates the interpretation of the results obtained. As a consequence, we limit this review to studies that (1) use mouse models that are deficient for, or overexpress, PPARs specifically in certain immune cells, (2) performed in vitro studies on immune cells deficient for, or overexpressing PPARs, and/or (3) used PPAR-specific (ant)agonists directly on (mouse or human) immune cells. In particular, we focus on studies concerning PPAR actions in macrophages and T cells, and how that impacts inflammatory disease (with a focus on metabolic and neurodegenerative diseases).

#### 3.2.1. Role of PPARs in Macrophages

All three PPAR family members have been shown to play a role in mouse macrophage polarization. PPARα, β, or γ activation was demonstrated to potentiate the polarization of mouse macrophages towards the anti-inflammatory M2 phenotype, while M2-type responses are compromised in the absence of PPARγ or β expression (effect of PPARα absence has not been studied) [50,51,52,53,54,55,56,57,58,59,60,61,62,63,64,65,66]. In human macrophages results are less clear-cut; while PPARγ activation has been shown to stimulate M2 polarization, PPARα or β activation did not seem to have any effect [67,68,69,70,71]. These anti-inflammatory actions of PPARs in macrophages have often been described to involve transrepression mechanisms involving NF-κB and AP-1 [51,53,60,61]. However, in line with the importance of metabolism in macrophage polarization (see Section 2.2 above), deletion of PPARγ in macrophages leads to reduced rates of β-oxidation of fatty acids, and consequently, these PPARγ-deficient macrophages are unable to clear the metabolic checkpoint required for full conversion to the alternative phenotype [50]. One mechanism through which PPARβ activation was proposed to exert its anti-inflammatory actions in macrophages involves the repressor BCL-6; unliganded PPARβ binds and sequesters BCL-6, and upon ligand binding, BCL-6 is released, and can repress transcription of pro-inflammatory target genes, including IL-1β, MCP-1, and matrix metalloproteinase 9 (MMP9) [72]. Based on this mechanism, PPARβ-deficient macrophages should exhibit an anti-inflammatory phenotype (BCL-6 would be free to repress pro-inflammatory genes). However, this is contradicted by two different studies that show that absence of PPARβ does not suppress pro-inflammatory responses during alternative activation of macrophages [66,73].

#### 3.2.2. Role of PPARs in T Cells

In T cells, PPARs have been shown to regulate survival, activation, and CD4^+^ T cell differentiation into the Th1, Th2, Th17, and Treg lineages [39]. PPARβ activation was shown to inhibit Th1 and Th17 polarization, and augment Th2 polarization, and the opposite was seen when PPARβ was deleted [74,75,76]. We have recently shown that activation or overexpression of PPARβ increases FAO in T cells [77]. Furthermore, using both in vivo and in vitro models, we demonstrated that PPARβ activation/overexpression inhibits thymic T cell development by decreasing proliferation of CD4^−^CD8^−^ double-negative stage 4 (DN4) thymocytes [77]. These results support a model where PPARβ activation/overexpression favors oxidation of fatty acids, instead of glucose, in developing T cells, thereby hampering the proliferative burst normally occurring at the DN4 stage of T cell development. As a consequence, the αβ T cells that are derived from DN4 thymocytes were dramatically decreased in peripheral lymphoid tissues, while the γδ T cell population remained untouched [77].

PPARγ activation was shown to impair T cell proliferation through an IL-2 dependent mechanism involving repression of nuclear factor of activated T cells (NFAT) [78,79]. Deletion of PPARγ in CD4^+^ T cells resulted in increased antigen-specific proliferation and overproduction of IFN-γ in response to IL-12, highlighting the importance of PPARγ expression in downregulating excessive Th1 responses [80]. Furthermore, PPARγ is highly expressed in both mouse and human Th2 cells, as opposed to other Th subsets, and although having a minor direct role in regulating Th2 differentiation, controls Th2 sensitivity to IL-33 and thus, has an impact on Th2 effector function [81]. However, PPARγ activation was reported to downregulate IL-4 production in T cells (through downregulation of NFAT) and expression of other Th2 cytokines (IL-5 and IL-13) was also reported to be decreased, as well as c-Maf, a Th2-specific transcription factor [82,83]. Together, these studies indicate that the effect of PPARγ activation on Th2 differentiation remains unclear.

Loss of PPARγ in Tregs has been shown to impair their ability to control effector CD4^+^ T cell responses while PPARγ activation in naïve CD4^+^ T cells enhanced induction of forkhead box P3 (FoxP3)^+^ inducible regulatory T cells [80,84,85]. Moreover, a recent study demonstrated that T cell-specific deletion of PPARγ leads to a specific reduction in GATA binding protein 3 (GATA3)-expressing Tregs [81]. In addition, a population of Tregs that highly expresses PPARγ has been identified in visceral adipose tissue, and Treg-specific deletion of PPARγ prevents accumulation of Tregs in visceral adipose tissue [86]. Furthermore, phosphorylation of serine 273 of PPARγ in Tregs changes the characteristic transcriptional signature of these Tregs [87]. Together, these studies suggest that PPARγ may contribute to the quality and quantity of Tregs.

In regard to Th17 differentiation, PPARγ activation was shown to have inhibitory effects while PPARγ deficiency led to increased Th17 differentiation [88]. Th17 differentiation depends on the transcription factor retinoic acid receptor (RAR)-related orphan receptor (ROR) γt, and the latter study by Klotz et al. demonstrated that under physiological conditions, the co-repressor silencing mediator of retinoid and thyroid hormone receptors (SMRT) is bound to the RORγt promoter and inhibits its transcription, and that PPARγ activation prevents removal of this corepressor complex, thereby suppressing RORγt expression and Th17 differentiation. It should also be mentioned that Klotz et al. did not observe an effect of PPARγ activation on Th1, Th2, or Treg T cell subsets, contradicting the above-mentioned studies.

#### 3.2.3. Gender-Specific Differences in the Role of PPARs in T Cells

One explanation for these contradicting results could be sex-specific roles of PPARs in T cells [89]. One of the first observations of gender differences in the role of PPARs in T cells was that T cells from male mice have increased expression of PPARα, compared to their female counterparts, and that the male sex hormone androgen has been suggested to regulate PPARα expression [90,91]. In the same study it was shown that PPARα-deficient T cells were predisposed to a Th1 response at the expense of Th2 function, and this was mediated by PPARα modulation of NF-κB and c-Jun activity. These results were recently confirmed by using a PPARα antagonist [92]. While PPARα expression is high in male T cells, PPARγ expression is high in female T cells [91], and the female sex hormone estrogen seems to influence expression of PPARγ [93]. As a result, the inhibitory role of PPARγ in T cell activation (see Section 3.2.2 above) is observed in female PPARγ-deficient T cells, but not in male T cells [94]. Similarly, PPARγ activation inhibits the differentiation of female Th1, Th2, and Th17 cells, whereas it specifically reduces only Th17-cell differentiation in males [95]. This provides a strong argument that, indeed, gender-specific differences in PPARγ expression in T cells could explain the contradictory results regarding the role of PPARγ in Th differentiation. PPARβ expression did not differ much when comparing male and female naïve and activated T cells [90].

Taken together, these studies demonstrate that the differential regulation of PPAR expression by sex hormones has an impact on the roles these receptors play in T cell biology. Furthermore, it cannot be excluded that contradictions in studies on the role of PPARs in macrophages, specifically the differences between mice and humans, could also potentially be the consequence of gender differences. Based on the importance of metabolism in immune cells (see Section 2 above), and the fact that most of the directly regulated PPAR target genes are involved in different aspects of fatty acid metabolism, it would seem obvious that the observed effects of PPARs on macrophage and T cell polarization/proliferation can be mechanistically explained by PPAR-induced changes in metabolism. However, this possibility was only rarely explored in the studies described above (and below).

## 4. Consequences of PPAR Actions in Immune Cells for Metabolic and Neurodegenerative Diseases

### 4.1. Metabolic Diseases

We focus here on the role of PPARs in immune cells in the context of atherosclerosis and obesity-associated inflammation and insulin resistance. Again, for reasons mentioned above (Section 3.2), studies using global knockouts or systemic treatments with agonists will not be discussed. Transplantation of PPARβ^−/−^ bone marrow into atherogenic diet-fed low-density lipoprotein receptor (LDLR)-deficient mice resulted in a reduction of aortic valve lesion surface compared to mice transplanted with wild type bone marrow [72]. Similarly, transplantation of bone marrow cells infected with lentivirus expressing selective microRNA (miRNA) targeting PPARβ into recipient LDLR^−/−^ mice resulted in reduction of atherosclerotic lesions, accompanied by a reduced presence of macrophages and expression of MCP-1 and MMP9 in the plaque [96]. This reduction of inflammation in absence of PPARβ in bone marrow cells is in line with the BCL-6 mechanistic model of PPARβ regulation of macrophage function. By contrast, transplantation of PPARγ^−/−^ bone marrow cells or conditional knockout of macrophage PPARγ increases atherosclerosis in both wild type and LDLR^−/−^ mice fed an atherogenic diet [97,98].

Two studies showed that macrophage-specific deletion of PPARγ predisposes mice to development of diet-induced obesity and insulin resistance [50,99]. Similar results were obtained when the effect of PPARβ-deficient bone marrow or macrophage-specific PPARβ^−/−^ on HFD-induced obesity and insulin resistance was studied [65,66]. However, one study found preserved glucose tolerance in mice transplanted with PPARγ^−/−^ or PPARβ^−/−^ bone marrow [100]. Since bone marrow-derived cells include T cells, some of the results outlined above could also be due to PPAR actions in T cells, even though the cited studies often interpreted them as macrophage specific. T cell-specific actions of PPARs, in the context of atherosclerosis or obesity-associated inflammation and insulin resistance, have largely been unexplored, with the exception of the role of PPARγ in adipose tissue Tregs in the latter. As mentioned already above (Section 3.2.2), PPARγ has been shown to be a crucial molecular orchestrator of visceral adipose tissue Treg accumulation, phenotype, and function [86,87]. Another area of PPAR research that deserves further exploration, not counting global knockout studies and systemic agonist treatment, is the specific role of PPARα in immune cells in the context of atherosclerosis and obesity-associated inflammation and insulin resistance.

### 4.2. Neurodegenerative Diseases

Even though neuroinflammation plays an important role in NDDs (outlined above in Section 1.2), and numerous studies have demonstrated beneficial effects of treatment with PPAR agonists in those pathologies, few studies have investigated how much PPAR actions in immune cells contribute to these positive effects observed [101,102]. In the context of Alzheimer’s disease, in vitro studies demonstrated that PPARγ agonists stimulated Aβ phagocytosis by rat primary microglia through induction of CD36 expression [103]. A similar study showed that PPARγ activation stimulated Aβ degradation by both primary mouse microglia and astrocytes, and that this involved a M1 to M2 shift for microglia [104]. Other in vitro studies revealed that pharmacological activation of PPARα attenuates the inflammatory responses of both primary mouse astrocytes and microglia [105,106]. The same group showed that PPARα activation in lipopolysaccharide (LPS)-treated microglia suppressed secretion of IL-12 family cytokines that are known to stimulate Th1 and Th17 differentiation [107]. Furthermore, they showed a similar decrease in IL-12 family cytokines in both microglia and astrocytes treated with PPARγ agonists [108,109], and PPARγ agonist inhibited the inflammatory response of those central nervous system (CNS) cells [110,111]. PPARγ activation in neuron–microglia co-cultures protected the neurons from damage induced by LPS-induced insults, by inhibiting microglia activation through interference with the NF-κB and AP-1 pathways [112]. In addition, PPARβ activation was shown to reduce LPS-stimulated nitric oxide (NO) production in enriched microglia and astrocyte cultures [113]. Likewise, PPARβ activation can also modulate radiation-induced oxidative stress and pro-inflammatory responses in microglia [114]. The latter was shown to occur through PPARβ interaction with the p65 subunit NF-κB.

Taken together, these in vitro cell culture studies demonstrate that PPAR activation reduces inflammation in both microglia and astrocytes and it is therefore likely that some (or most) of the beneficial effects observed with PPAR activation in NDDs are the consequence of anti-inflammatory PPAR actions in these cells. However, to study the specific role of microglial and astrocyte PPARs in NDDs in an in vivo context, it would be of great interest to overexpress or knockout PPARs in a cell-type specific fashion using CX3C chemokine receptor 1 (CX3CR1)-Cre or glial fibrillary acid protein (GFAP)-Cre mice, respectively. Even though the CX3CR1-Cre approach will also affect other CX3CR1-expressing myeloid cell populations, these types of studies would still be very informative.

## 5. Conclusions

**Figure 2:**
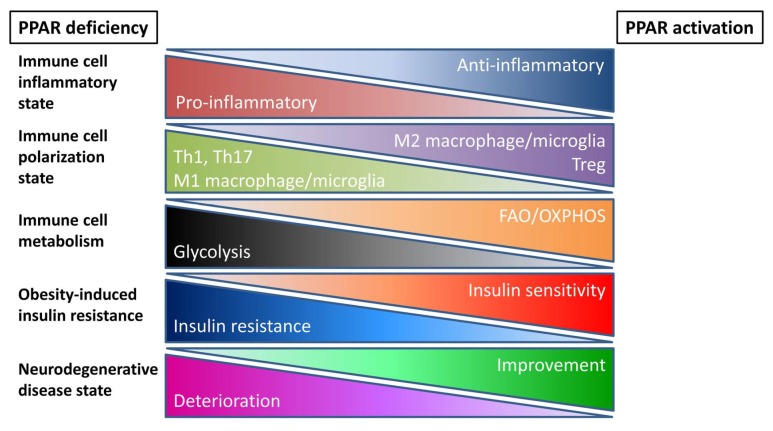
Effects of peroxisome proliferator-activated receptor (PPAR) deficiency or activation on immune cell properties and metabolic and neurodegenerative disease states. Despite some contradictory results (perhaps due to gender differences), the overall impression we deduce from the literature is that PPAR activation has anti-inflammatory effects on immune cells by stimulating the polarization of these cells towards more anti-inflammatory subsets. Perhaps the switch towards FAO/OXPHOS (fatty acid oxidation/oxidative phosphorylation) metabolism induced by PPAR activation plays an important role in this shift towards anti-inflammatory immune cell subsets. By contrast, PPAR deficiency has often been shown to have the opposite effects. Together, these PPAR-regulated properties of immune cells might contribute to the severity of the disease state both in metabolic diseases (e.g., obesity-induced insulin resistance) and neurodegenerative disorders NDDs.

In summary, inflammation has been shown to be a common denominator in both metabolic syndrome and NDDs, and targeting this inflammation from a therapeutic standpoint could potentially have beneficial consequences for both pathologies. Based on the anti-inflammatory effects that have been attributed to PPARs, and the roles that have been described for these receptors in regard to immune cell functions, activating these receptors, specifically in immune cells, could be considered as such a therapeutic approach (see Figure 2). This immune cell-specific approach could circumvent certain adverse effects that have been observed in the past with systemic treatments with PPAR agonists. However, before pursuing such an ambitious goal, several insufficiently explored questions in PPAR research should be further addressed. While many studies strongly suggest that beneficial effects of PPAR activation in the context of metabolic syndrome and NDDs can be explained by anti-inflammatory effects, direct proof of an important role for PPAR-induced changes in immune cell function is often lacking. This missing proof could be supplied by studying the effects of immune cell-specific deficiency or overexpression of PPARs in the context of metabolic disease and NDD mouse models. It is important that potential gender-specific differences should be taken into account while conducting these types of studies. Lastly, PPAR-induced metabolic changes should be more often considered/explored as a mechanistic explanation of the regulatory functions that are attributed to these nuclear receptors in immune cells. 

## Abbreviations

AP-1	activator protein-1
Aβ	amyloid β-peptide
BCL-6	B-cell lymphoma 6
Bregs	regulatory B cells 6
CNS	central nervous system
CPT1a	carnitine palmitoyltransferase Ia
CX3CR1	CX3C chemokine receptor 1
DC	dendritic cells
FAO	fatty acid oxidation
FAT	fatty acid translocase
FoxP3	forkhead box P3
GATA3	GATA binding protein 3
GFAP	glial fibrillary acid protein
GLUT1	glucose transporter 1
HTT	huntingtin
IFNγ	interferon γ
IL	interleukin
ILC2s	type 2 innate lymphoid cells
LDLR	low-density lipoprotein receptor
LPS	lipopolysaccharide
MAPK	mitogen-activated protein kinase
MCP-1	monocyte chemoattractant protein 1
miRNA	microRNA
MMP9	matrix metalloproteinase-9
NDDs	neurodegenerative disorders
NF-κB	nuclear factor-κB
NFAT	nuclear factor of activated T cells
NK	natural killer cells
NO	nitric oxide
OXPHOS	oxidative phosphorylation
PPAR	peroxisome proliferator-activated receptor
PPREs	peroxisome proliferator response elements
RAR	retinoic acid receptor
ROR	related orphan receptor
RXR	retinoid X receptor
SMRT	silencing mediator of retinoid and thyroid hormone receptors
Th	T helper
TNFα	tumor necrosis factor alpha
Tregs	regulatory T cells

## References

[1] Hotamisligil G.S. (2006). Inflammation and metabolic disorders. Nature.

[2] McLaughlin T., Ackerman S.E., Shen L., Engleman E. (2017). Role of innate and adaptive immunity in obesity-associated metabolic disease. J. Clin. Investig..

[3] Tabas I., Lichtman A.H. (2017). Monocyte-Macrophages and T Cells in Atherosclerosis. Immunity.

[4] De Candia P., Matarese G. (2017). Leptin and ghrelin: Sewing metabolism onto neurodegeneration. Neuropharmacology.

[5] Abbott R.D., Ross G.W., White L.R., Nelson J.S., Masaki K.H., Tanner C.M., Curb J.D., Blanchette P.L., Popper J.S., Petrovitch H. (2002). Midlife adiposity and the future risk of Parkinson’s disease. Neurology.

[6] Kivipelto M., Ngandu T., Fratiglioni L., Viitanen M., Kareholt I., Winblad B., Helkala E.L., Tuomilehto J., Soininen H., Nissinen A. (2005). Obesity and vascular risk factors at midlife and the risk of dementia and Alzheimer disease. Arch. Neurol..

[7] Xu W.L., Atti A.R., Gatz M., Pedersen N.L., Johansson B., Fratiglioni L. (2011). Midlife overweight and obesity increase late-life dementia risk: A population-based twin study. Neurology.

[8] Colman R.J., Anderson R.M., Johnson S.C., Kastman E.K., Kosmatka K.J., Beasley T.M., Allison D.B., Cruzen C., Simmons H.A., Kemnitz J.W. (2009). Caloric restriction delays disease onset and mortality in rhesus monkeys. Science.

[9] Guerrero-Garcia J.J., Carrera-Quintanar L., Lopez-Roa R.I., Marquez-Aguirre A.L., Rojas-Mayorquin A.E., Ortuno-Sahagun D. (2016). Multiple Sclerosis and Obesity: Possible Roles of Adipokines. Mediat. Inflamm..

[10] Ransohoff R.M. (2016). How neuroinflammation contributes to neurodegeneration. Science.

[11] Jais A., Bruning J.C. (2017). Hypothalamic inflammation in obesity and metabolic disease. J. Clin. Investig..

[12] Maldonado-Ruiz R., Montalvo-Martinez L., Fuentes-Mera L., Camacho A. (2017). Microglia activation due to obesity programs metabolic failure leading to type two diabetes. Nutr. Diabetes.

[13] Calegari V.C., Torsoni A.S., Vanzela E.C., Araujo E.P., Morari J., Zoppi C.C., Sbragia L., Boschero A.C., Velloso L.A. (2016). Inflammation of the hypothalamus leads to defective pancreatic islet function. J. Biol. Chem..

[14] Scherer T., Lindtner C., Zielinski E., O’Hare J., Filatova N., Buettner C. (2012). Short term voluntary overfeeding disrupts brain insulin control of adipose tissue lipolysis. J. Biol. Chem..

[15] Crotti A., Glass C.K. (2015). The choreography of neuroinflammation in Huntington’s disease. Trends Immunol..

[16] Frauwirth K.A., Riley J.L., Harris M.H., Parry R.V., Rathmell J.C., Plas D.R., Elstrom R.L., June C.H., Thompson C.B. (2002). The CD28 signaling pathway regulates glucose metabolism. Immunity.

[17] Caro-Maldonado A., Wang R., Nichols A.G., Kuraoka M., Milasta S., Sun L.D., Gavin A.L., Abel E.D., Kelsoe G., Green D.R. (2014). Metabolic reprogramming is required for antibody production that is suppressed in anergic but exaggerated in chronically BAFF-exposed B cells. J. Immunol..

[18] Macintyre A.N., Gerriets V.A., Nichols A.G., Michalek R.D., Rudolph M.C., Deoliveira D., Anderson S.M., Abel E.D., Chen B.J., Hale L.P. (2014). The glucose transporter Glut1 is selectively essential for CD4 T cell activation and effector function. Cell Metab..

[19] Doughty C.A., Bleiman B.F., Wagner D.J., Dufort F.J., Mataraza J.M., Roberts M.F., Chiles T.C. (2006). Antigen receptor-mediated changes in glucose metabolism in B lymphocytes: Role of phosphatidylinositol 3-kinase signaling in the glycolytic control of growth. Blood.

[20] Wang R., Dillon C.P., Shi L.Z., Milasta S., Carter R., Finkelstein D., McCormick L.L., Fitzgerald P., Chi H., Munger J. (2011). The transcription factor Myc controls metabolic reprogramming upon T lymphocyte activation. Immunity.

[21] Le A., Lane A.N., Hamaker M., Bose S., Gouw A., Barbi J., Tsukamoto T., Rojas C.J., Slusher B.S., Zhang H. (2012). Glucose-independent glutamine metabolism via TCA cycling for proliferation and survival in B cells. Cell Metab..

[22] Michalek R.D., Gerriets V.A., Jacobs S.R., Macintyre A.N., MacIver N.J., Mason E.F., Sullivan S.A., Nichols A.G., Rathmell J.C. (2011). Cutting edge: Distinct glycolytic and lipid oxidative metabolic programs are essential for effector and regulatory CD4^+^ T cell subsets. J. Immunol..

[23] Shi L.Z., Wang R., Huang G., Vogel P., Neale G., Green D.R., Chi H. (2011). HIF1α-dependent glycolytic pathway orchestrates a metabolic checkpoint for the differentiation of TH17 and Treg cells. J. Exp. Med..

[24] van der Windt G.J., Everts B., Chang C.H., Curtis J.D., Freitas T.C., Amiel E., Pearce E.J., Pearce E.L. (2012). Mitochondrial respiratory capacity is a critical regulator of CD8^+^ T cell memory development. Immunity.

[25] Rodriguez-Espinosa O., Rojas-Espinosa O., Moreno-Altamirano M.M., Lopez-Villegas E.O., Sanchez-Garcia F.J. (2015). Metabolic requirements for neutrophil extracellular traps formation. Immunology.

[26] Lane T.A., Lamkin G.E. (1984). A reassessment of the energy requirements for neutrophil migration: Adenosine triphosphate depletion enhances chemotaxis. Blood.

[27] Phong B., Avery L., Menk A.V., Delgoffe G.M., Kane L.P. (2017). Cutting Edge: Murine Mast Cells Rapidly Modulate Metabolic Pathways Essential for Distinct Effector Functions. J. Immunol..

[28] Sumbayev V.V., Nicholas S.A., Streatfield C.L., Gibbs B.F. (2009). Involvement of hypoxia-inducible factor-1 HiF(1α) in IgE-mediated primary human basophil responses. Eur. J. Immunol..

[29] Everts B., Amiel E., Huang S.C., Smith A.M., Chang C.H., Lam W.Y., Redmann V., Freitas T.C., Blagih J., van der Windt G.J. (2014). TLR-driven early glycolytic reprogramming via the kinases TBK1-IKKvarepsilon supports the anabolic demands of dendritic cell activation. Nat. Immunol..

[30] Huang S.C., Everts B., Ivanova Y., O’Sullivan D., Nascimento M., Smith A.M., Beatty W., Love-Gregory L., Lam W.Y., O’Neill C.M. (2014). Cell-intrinsic lysosomal lipolysis is essential for alternative activation of macrophages. Nat. Immunol..

[31] Namgaladze D., Lips S., Leiker T.J., Murphy R.C., Ekroos K., Ferreiros N., Geisslinger G., Brune B. (2014). Inhibition of macrophage fatty acid β-oxidation exacerbates palmitate-induced inflammatory and endoplasmic reticulum stress responses. Diabetologia.

[32] Varga T., Czimmerer Z., Nagy L. (2011). PPARs are a unique set of fatty acid regulated transcription factors controlling both lipid metabolism and inflammation. Biochim. Biophys. Acta.

[33] Zoete V., Grosdidier A., Michielin O. (2007). Peroxisome proliferator-activated receptor structures: Ligand specificity, molecular switch and interactions with regulators. Biochim. Biophys. Acta.

[34] Ricote M., Glass C.K. (2007). PPARs and molecular mechanisms of transrepression. Biochim. Biophys. Acta.

[35] Neels J.G., Grimaldi P.A. (2014). Physiological functions of peroxisome proliferator-activated receptor β. Physiol. Rev..

[36] Fuentes E., Guzman-Jofre L., Moore-Carrasco R., Palomo I. (2013). Role of PPARs in inflammatory processes associated with metabolic syndrome. Mol. Med. Rep..

[37] Gervois P., Mansouri R.M. (2012). PPARα as a therapeutic target in inflammation-associated diseases. Expert Opin. Ther. Targets.

[38] Wahli W., Michalik L. (2012). PPARs at the crossroads of lipid signaling and inflammation. Trends Endocrinol. Metab. TEM.

[39] Choi J.M., Bothwell A.L. (2012). The nuclear receptor PPARs as important regulators of T-cell functions and autoimmune diseases. Mol. Cells.

[40] Bishop-Bailey D., Bystrom J. (2009). Emerging roles of peroxisome proliferator-activated receptor-β/δ in inflammation. Pharmacol. Ther..

[41] Hong C., Tontonoz P. (2008). Coordination of inflammation and metabolism by PPAR and LXR nuclear receptors. Curr. Opin. Genet. Dev..

[42] Straus D.S., Glass C.K. (2007). Anti-inflammatory actions of PPAR ligands: New insights on cellular and molecular mechanisms. Trends Immunol..

[43] Szeles L., Torocsik D., Nagy L. (2007). PPARγ in immunity and inflammation: Cell types and diseases. Biochim. Biophys. Acta.

[44] Rizzo G., Fiorucci S. (2006). PPARs and other nuclear receptors in inflammation. Curr. Opin. Pharmacol..

[45] Kostadinova R., Wahli W., Michalik L. (2005). PPARs in diseases: Control mechanisms of inflammation. Curr. Med. Chem..

[46] Moraes L.A., Piqueras L., Bishop-Bailey D. (2006). Peroxisome proliferator-activated receptors and inflammation. Pharmacol. Ther..

[47] Chinetti G., Fruchart J.C., Staels B. (2003). Peroxisome proliferator-activated receptors and inflammation: From basic science to clinical applications. Int. J. Obes..

[48] Cabrero A., Laguna J.C., Vazquez M. (2002). Peroxisome proliferator-activated receptors and the control of inflammation. Curr. Drug Targets Inflamm. Allergy.

[49] Chinetti G., Fruchart J.C., Staels B. (2000). Peroxisome proliferator-activated receptors (PPARs): Nuclear receptors at the crossroads between lipid metabolism and inflammation. Inflamm. Res..

[50] Odegaard J.I., Ricardo-Gonzalez R.R., Goforth M.H., Morel C.R., Subramanian V., Mukundan L., Red Eagle A., Vats D., Brombacher F., Ferrante A.W. (2007). Macrophage-specific PPARγ controls alternative activation and improves insulin resistance. Nature.

[51] Penas F., Mirkin G.A., Vera M., Cevey A., Gonzalez C.D., Gomez M.I., Sales M.E., Goren N.B. (2015). Treatment in vitro with PPARα and PPARγ ligands drives M1-to-M2 polarization of macrophages from T. cruzi-infected mice. Biochim. Biophys. Acta.

[52] Gallardo-Soler A., Gomez-Nieto C., Campo M.L., Marathe C., Tontonoz P., Castrillo A., Corraliza I. (2008). Arginase I induction by modified lipoproteins in macrophages: A peroxisome proliferator-activated receptor-γ/δ-mediated effect that links lipid metabolism and immunity. Mol. Endocrinol..

[53] Luo W., Xu Q., Wang Q., Wu H., Hua J. (2017). Effect of modulation of PPAR-γ activity on Kupffer cells M1/M2 polarization in the development of non-alcoholic fatty liver disease. Sci. Rep..

[54] Zhong X., Liu H. (2018). Honokiol attenuates diet-induced non-alcoholic steatohepatitis by regulating macrophage polarization through activating peroxisome proliferator-activated receptor γ. J. Gastroenterol. Hepatol..

[55] Li C., Ying W., Huang Z., Brehm T., Morin A., Vella A.T., Zhou B. (2017). IRF6 Regulates Alternative Activation by Suppressing PPARγ in Male Murine Macrophages. Endocrinology.

[56] Bermudez B., Dahl T.B., Medina I., Groeneweg M., Holm S., Montserrat-de la Paz S., Rousch M., Otten J., Herias V., Varela L.M. (2017). Leukocyte Overexpression of Intracellular NAMPT Attenuates Atherosclerosis by Regulating PPARγ-Dependent Monocyte Differentiation and Function. Arterioscler. Thromb. Vasc. Biol..

[57] Tikhanovich I., Zhao J., Olson J., Adams A., Taylor R., Bridges B., Marshall L., Roberts B., Weinman S.A. (2017). Protein arginine methyltransferase 1 modulates innate immune responses through regulation of peroxisome proliferator-activated receptor γ-dependent macrophage differentiation. J. Biol. Chem..

[58] Assuncao L.S., Magalhaes K.G., Carneiro A.B., Molinaro R., Almeida P.E., Atella G.C., Castro-Faria-Neto H.C., Bozza P.T. (2017). Schistosomal-derived lysophosphatidylcholine triggers M2 polarization of macrophages through PPARγ dependent mechanisms. Biochim. Biophys. Acta.

[59] Zhang M., Zhou Z., Wang J., Li S. (2016). MiR-130b promotes obesity associated adipose tissue inflammation and insulin resistance in diabetes mice through alleviating M2 macrophage polarization via repression of PPAR-γ. Immunol. Lett..

[60] Feng X., Weng D., Zhou F., Owen Y.D., Qin H., Zhao J., WenYu, Huang Y., Chen J., Fu H., Yang N. (2016). Activation of PPARγ by a natural flavonoid modulator, apigenin ameliorates obesity-related inflammation via regulation of macrophage polarization. EBioMedicine.

[61] Deng X., Zhang P., Liang T., Deng S., Chen X., Zhu L. (2015). Ovarian cancer stem cells induce the M2 polarization of macrophages through the PPAγ and NF-κB pathways. Int. J. Mol. Med..

[62] Zhang X., Zhou M., Guo Y., Song Z., Liu B. (2015). 1,25-Dihydroxyvitamin D_3_ Promotes High Glucose-Induced M1 Macrophage Switching to M2 via the VDR-PPARγ Signaling Pathway. Biomed. Res. Int..

[63] Chang H.Y., Lee H.N., Kim W., Surh Y.J. (2015). Docosahexaenoic acid induces M2 macrophage polarization through peroxisome proliferator-activated receptor γ activation. Life Sci..

[64] Feng X., Qin H., Shi Q., Zhang Y., Zhou F., Wu H., Ding S., Niu Z., Lu Y., Shen P. (2014). Chrysin attenuates inflammation by regulating M1/M2 status via activating PPARγ. Biochem. Pharmacol..

[65] Kang K., Reilly S.M., Karabacak V., Gangl M.R., Fitzgerald K., Hatano B., Lee C.H. (2008). Adipocyte-derived Th2 cytokines and myeloid PPARδ regulate macrophage polarization and insulin sensitivity. Cell Metab..

[66] Odegaard J.I., Ricardo-Gonzalez R.R., Red Eagle A., Vats D., Morel C.R., Goforth M.H., Subramanian V., Mukundan L., Ferrante A.W., Chawla A. (2008). Alternative M2 activation of Kupffer cells by PPARδ ameliorates obesity-induced insulin resistance. Cell Metab..

[67] Bouhlel M.A., Brozek J., Derudas B., Zawadzki C., Jude B., Staels B., Chinetti-Gbaguidi G. (2009). Unlike PPARγ, PPARα or PPARβ/δ activation does not promote human monocyte differentiation toward alternative macrophages. Biochem. Biophys. Res. Commun..

[68] Bouhlel M.A., Derudas B., Rigamonti E., Dievart R., Brozek J., Haulon S., Zawadzki C., Jude B., Torpier G., Marx N. (2007). PPARγ activation primes human monocytes into alternative M2 macrophages with anti-inflammatory properties. Cell Metab..

[69] Zhang T., Shao B., Liu G.A. (2017). Rosuvastatin promotes the differentiation of peripheral blood monocytes into M2 macrophages in patients with atherosclerosis by activating PPAR-γ. Eur. Rev. Med. Pharmacol. Sci..

[70] Zizzo G., Cohen P.L. (2015). The PPAR-γ antagonist GW9662 elicits differentiation of M2c-like cells and upregulation of the MerTK/Gas6 axis: A key role for PPAR-γ in human macrophage polarization. J. Inflamm..

[71] Zhang O., Zhang J. (2015). Atorvastatin promotes human monocyte differentiation toward alternative M2 macrophages through p38 mitogen-activated protein kinase-dependent peroxisome proliferator-activated receptor γ activation. Int. Immunopharmacol..

[72] Lee C.H., Chawla A., Urbiztondo N., Liao D., Boisvert W.A., Evans R.M., Curtiss L.K. (2003). Transcriptional repression of atherogenic inflammation: Modulation by PPARδ. Science.

[73] Mukundan L., Odegaard J.I., Morel C.R., Heredia J.E., Mwangi J.W., Ricardo-Gonzalez R.R., Goh Y.P., Eagle A.R., Dunn S.E., Awakuni J.U. (2009). PPAR-δ senses and orchestrates clearance of apoptotic cells to promote tolerance. Nat. Med..

[74] Kanakasabai S., Chearwae W., Walline C.C., Iams W., Adams S.M., Bright J.J. (2010). Peroxisome proliferator-activated receptor δ agonists inhibit T helper type 1 (Th1) and Th17 responses in experimental allergic encephalomyelitis. Immunology.

[75] Kanakasabai S., Walline C.C., Chakraborty S., Bright J.J. (2011). PPARδ deficient mice develop elevated Th1/Th17 responses and prolonged experimental autoimmune encephalomyelitis. Brain Res..

[76] Dunn S.E., Bhat R., Straus D.S., Sobel R.A., Axtell R., Johnson A., Nguyen K., Mukundan L., Moshkova M., Dugas J.C. (2010). Peroxisome proliferator-activated receptor δ limits the expansion of pathogenic Th cells during central nervous system autoimmunity. J. Exp. Med..

[77] Mothe-Satney I., Murdaca J., Sibille B., Rousseau A.S., Squillace R., Le Menn G., Rekima A., Larbret F., Pele J., Verhasselt V. (2016). A role for Peroxisome Proliferator-Activated Receptor Beta in T cell development. Sci. Rep..

[78] Clark R.B., Bishop-Bailey D., Estrada-Hernandez T., Hla T., Puddington L., Padula S.J. (2000). The nuclear receptor PPAR γ and immunoregulation: PPAR γ mediates inhibition of helper T cell responses. J. Immunol..

[79] Yang X.Y., Wang L.H., Chen T., Hodge D.R., Resau J.H., DaSilva L., Farrar W.L. (2000). Activation of human T lymphocytes is inhibited by peroxisome proliferator-activated receptor γ (PPARγ) agonists. PPARγ co-association with transcription factor NFAT. J. Biol. Chem..

[80] Hontecillas R., Bassaganya-Riera J. (2007). Peroxisome proliferator-activated receptor γ is required for regulatory CD4^+^ T cell-mediated protection against colitis. J. Immunol..

[81] Nobs S.P., Natali S., Pohlmeier L., Okreglicka K., Schneider C., Kurrer M., Sallusto F., Kopf M. (2017). PPARγ in dendritic cells and T cells drives pathogenic type-2 effector responses in lung inflammation. J. Exp. Med..

[82] Chung S.W., Kang B.Y., Kim T.S. (2003). Inhibition of interleukin-4 production in CD4^+^ T cells by peroxisome proliferator-activated receptor-γ (PPAR-γ) ligands: Involvement of physical association between PPAR-γ and the nuclear factor of activated T cells transcription factor. Mol. Pharmacol..

[83] Won H.Y., Min H.J., Ahn J.H., Yoo S.E., Bae M.A., Hong J.H., Hwang E.S. (2010). Anti-allergic function and regulatory mechanisms of KR62980 in allergen-induced airway inflammation. Biochem. Pharmacol..

[84] Guri A.J., Mohapatra S.K., Horne W.T., Hontecillas R., Bassaganya-Riera J. (2010). The role of T cell PPAR γ in mice with experimental inflammatory bowel disease. BMC Gastroenterol..

[85] Wohlfert E.A., Nichols F.C., Nevius E., Clark R.B. (2007). Peroxisome proliferator-activated receptor γ (PPARγ) and immunoregulation: Enhancement of regulatory T cells through PPARγ-dependent and -independent mechanisms. J. Immunol..

[86] Cipolletta D., Feuerer M., Li A., Kamei N., Lee J., Shoelson S.E., Benoist C., Mathis D. (2012). PPAR-γ is a major driver of the accumulation and phenotype of adipose tissue Treg cells. Nature.

[87] Cipolletta D., Cohen P., Spiegelman B.M., Benoist C., Mathis D. (2015). Appearance and disappearance of the mRNA signature characteristic of Treg cells in visceral adipose tissue: Age, diet, and PPARγ effects. Proc. Natl. Acad. Sci. USA.

[88] Klotz L., Burgdorf S., Dani I., Saijo K., Flossdorf J., Hucke S., Alferink J., Nowak N., Beyer M., Mayer G. (2009). The nuclear receptor PPAR γ selectively inhibits Th17 differentiation in a T cell-intrinsic fashion and suppresses CNS autoimmunity. J. Exp. Med..

[89] Park H.J., Choi J.M. (2017). Sex-specific regulation of immune responses by PPARs. Exp. Mol. Med..

[90] Dunn S.E., Ousman S.S., Sobel R.A., Zuniga L., Baranzini S.E., Youssef S., Crowell A., Loh J., Oksenberg J., Steinman L. (2007). Peroxisome proliferator-activated receptor (PPAR)α expression in T cells mediates gender differences in development of T cell-mediated autoimmunity. J. Exp. Med..

[91] Zhang M.A., Rego D., Moshkova M., Kebir H., Chruscinski A., Nguyen H., Akkermann R., Stanczyk F.Z., Prat A., Steinman L. (2012). Peroxisome proliferator-activated receptor (PPAR)α and -γ regulate IFNγ and IL-17A production by human T cells in a sex-specific way. Proc. Natl. Acad. Sci. USA.

[92] Zhang M.A., Ahn J.J., Zhao F.L., Selvanantham T., Mallevaey T., Stock N., Correa L., Clark R., Spaner D., Dunn S.E. (2015). Antagonizing Peroxisome Proliferator-Activated Receptor α Activity Selectively Enhances Th1 Immunity in Male Mice. J. Immunol..

[93] Park H.J., Park H.S., Lee J.U., Bothwell A.L., Choi J.M. (2016). Gender-specific differences in PPARγ regulation of follicular helper T cell responses with estrogen. Sci. Rep..

[94] Park H.J., Kim D.H., Choi J.Y., Kim W.J., Kim J.Y., Senejani A.G., Hwang S.S., Kim L.K., Tobiasova Z., Lee G.R. (2014). PPARγ negatively regulates T cell activation to prevent follicular helper T cells and germinal center formation. PLoS ONE.

[95] Park H.J., Park H.S., Lee J.U., Bothwell A.L., Choi J.M. (2016). Sex-Based Selectivity of PPARγ Regulation in Th1, Th2, and Th17 Differentiation. Int. J. Mol. Sci..

[96] Li G., Chen C., Laing S.D., Ballard C., Biju K.C., Reddick R.L., Clark R.A., Li S. (2016). Hematopoietic knockdown of PPARδ reduces atherosclerosis in LDLR^−/−^ mice. Gene Ther..

[97] Babaev V.R., Yancey P.G., Ryzhov S.V., Kon V., Breyer M.D., Magnuson M.A., Fazio S., Linton M.F. (2005). Conditional knockout of macrophage PPARγ increases atherosclerosis in C57BL/6 and low-density lipoprotein receptor-deficient mice. Arterioscler. Thromb. Vasc. Boil..

[98] Chawla A., Boisvert W.A., Lee C.H., Laffitte B.A., Barak Y., Joseph S.B., Liao D., Nagy L., Edwards P.A., Curtiss L.K. (2001). A PPAR γ-LXR-ABCA1 pathway in macrophages is involved in cholesterol efflux and atherogenesis. Mol. Cell.

[99] Hevener A.L., Olefsky J.M., Reichart D., Nguyen M.T., Bandyopadyhay G., Leung H.Y., Watt M.J., Benner C., Febbraio M.A., Nguyen A.K. (2007). Macrophage PPAR γ is required for normal skeletal muscle and hepatic insulin sensitivity and full antidiabetic effects of thiazolidinediones. J. Clin. Investig..

[100] Marathe C., Bradley M.N., Hong C., Chao L., Wilpitz D., Salazar J., Tontonoz P. (2009). Preserved glucose tolerance in high-fat-fed C57BL/6 mice transplanted with PPARγ^−/−^, PPARδ^−/−^, PPARγδ^−/−^, or LXRαβ^−/−^ bone marrow. J. Lipid Res..

[101] Skerrett R., Malm T., Landreth G. (2014). Nuclear receptors in neurodegenerative diseases. Neurobiol. Dis..

[102] Iglesias J., Morales L., Barreto G.E. (2017). Metabolic and Inflammatory Adaptation of Reactive Astrocytes: Role of PPARs. Mol. Neurobiol..

[103] Yamanaka M., Ishikawa T., Griep A., Axt D., Kummer M.P., Heneka M.T. (2012). PPARγ/RXRα-induced and CD36-mediated microglial amyloid-β phagocytosis results in cognitive improvement in amyloid precursor protein/presenilin 1 mice. J. Neurosci..

[104] Mandrekar-Colucci S., Karlo J.C., Landreth G.E. (2012). Mechanisms underlying the rapid peroxisome proliferator-activated receptor-γ-mediated amyloid clearance and reversal of cognitive deficits in a murine model of Alzheimer’s disease. J. Neurosci..

[105] Xu J., Chavis J.A., Racke M.K., Drew P.D. (2006). Peroxisome proliferator-activated receptor-α and retinoid X receptor agonists inhibit inflammatory responses of astrocytes. J. Neuroimmunol..

[106] Xu J., Storer P.D., Chavis J.A., Racke M.K., Drew P.D. (2005). Agonists for the peroxisome proliferator-activated receptor-α and the retinoid X receptor inhibit inflammatory responses of microglia. J. Neurosci. Res..

[107] Xu J., Racke M.K., Drew P.D. (2007). Peroxisome proliferator-activated receptor-α agonist fenofibrate regulates IL-12 family cytokine expression in the CNS: Relevance to multiple sclerosis. J. Neurochem..

[108] Xu J., Drew P.D. (2007). Peroxisome proliferator-activated receptor-γ agonists suppress the production of IL-12 family cytokines by activated glia. J. Immunol..

[109] Xu J., Barger S.W., Drew P.D. (2008). The PPAR-γ Agonist 15-Deoxy-Delta-Prostaglandin J(2) Attenuates Microglial Production of IL-12 Family Cytokines: Potential Relevance to Alzheimer’s Disease. PPAR Res..

[110] Storer P.D., Xu J., Chavis J., Drew P.D. (2005). Peroxisome proliferator-activated receptor-γ agonists inhibit the activation of microglia and astrocytes: Implications for multiple sclerosis. J. Neuroimmunol..

[111] Storer P.D., Xu J., Chavis J.A., Drew P.D. (2005). Cyclopentenone prostaglandins PGA2 and 15-deoxy-δ12,14 PGJ2 suppress activation of murine microglia and astrocytes: Implications for multiple sclerosis. J. Neurosci. Res..

[112] Xing B., Liu M., Bing G. (2007). Neuroprotection with pioglitazone against LPS insult on dopaminergic neurons may be associated with its inhibition of NF-κB and JNK activation and suppression of COX-2 activity. J. Neuroimmunol..

[113] Polak P.E., Kalinin S., Dello Russo C., Gavrilyuk V., Sharp A., Peters J.M., Richardson J., Willson T.M., Weinberg G., Feinstein D.L. (2005). Protective effects of a peroxisome proliferator-activated receptor-β/δ agonist in experimental autoimmune encephalomyelitis. J. Neuroimmunol..

[114] Schnegg C.I., Kooshki M., Hsu F.C., Sui G., Robbins M.E. (2012). PPARδ prevents radiation-induced proinflammatory responses in microglia via transrepression of NF-κB and inhibition of the PKCα/MEK1/2/ERK1/2/AP-1 pathway. Free Radic. Biol. Med..

